# Effects of land use change on population survival of three wild rice species in China since 2001

**DOI:** 10.3389/fpls.2022.951903

**Published:** 2022-09-06

**Authors:** Hao Chen, Shanshan Dong, Zhizhou He, Yuhong Chen, Defeng Tian, Yan Liu, Yuguo Wang, Wenju Zhang, Linfeng Li, Ji Yang, Zhiping Song

**Affiliations:** ^1^The Ministry of Education Key Laboratory for Biodiversity Science and Ecological Engineering, Institute of Biodiversity Science, Institute of Botany, Fudan University, Shanghai, China; ^2^Nanjing Institute of Environmental Sciences of the Ministry of Ecology and Environment, Nanjing, China

**Keywords:** conservation, habitat, land use, community, wild rice, wild population

## Abstract

Land use change stemming from human activities, particularly cropland expansion, heavily threatens the survival of crop wild relatives that usually occur nearby or scatter in farming systems. Understanding the impacts of land use change on wild populations is critical in forming the conservation decision-making of wild relatives. Based on the investigations on the population survival of three wild rice species (*Oryza rufipogon*, *O. officinalis*, and *O. granulata*) in China over the past 40 years (1978–2019), the effect of land use change during the past 20 years (2001–2019) on the natural populations of the three species was examined using the land use type data of satellite-based Earth observations (data from GlobCover). From 1978 to 2019, the number of populations (distribution sites) of the three wild rice species had decreased by 65–87%, mainly because of the habitat destruction or disappearance caused by human-induced land use change. The three wild rice species display different habitat preferences, resulting in specific land use types surrounding their populations. In the recent 20 years, although the surrounding community composition of the wild rice population has been relatively stable, the surrounding vegetation cover area of the survived populations was significantly more extensive than that of the extinct ones (*p* < 0.05). These findings suggest that habitat vegetation plays a “biological barrier” role in the survival of wild populations through resisting or mitigating the disturbing impact of land use change on wild populations. This study provides not only direct guidelines for the conservation of wild rice but also new insights into the mechanisms underlying the influence of land use change on wild populations.

## Introduction

Crop wild relatives (CWRs) are the most important genetic resources for germplasm innovation and crop improvement (Xiao et al., 1996; Hajjar and Hodgkin, 2007; Feuillet et al., 2008). They play an important role in raising the yield and quality of grains and other economic crops (Vitousek et al., 1997). However, CWRs have been seriously threatened by human activities around the world. On the one hand, people need to increase land use to supply food for the rapidly growing population; this increased land use involves land exploitation and agricultural expansion that inevitably leads to the destruction or even loss of natural habitats, threatening the survival of wild populations. On the other hand, CWRs have habitat requirements similar to those of domesticated crops. Thus, they usually scatter in agricultural systems, and their habitats tend to be directly exploited or disturbed by agricultural activities, such as the use of agrochemicals (pesticides and fertilizers), grazing, and the introduction of nonnative competitors, resulting in population decline or even extinction of CWRs (Newbold et al., 2015; Pimm et al., 2018; Nicholson et al., 2019). Therefore, land use change is the main threat to CWRs survival (Davies et al., 2006). Evaluating the effect of land use change on wild populations is critical for CWRs conservation.

As an important genetic resources, CWRs have attracted the attention of conservationists and plant breeders. However, little is known about how land use change impacts the survival of their wild populations. Several studies that focused on the population genetics of CWRs suggested that the genetic population decline is associated with habitat fragmentation due to human activities (Ceballos et al., 2017). Consequently, *in situ* conservation strategies have been established to maintain population genetic variations, thereby protecting the evolutionary potential of the wild population. The survival of wild populations mainly depends on the habitat quality and the stability of the natural community surrounding wild populations (Blowes et al., 2019; Newbold et al., 2020). Community stability is highly related to landscape structure and heterogeneity. However, land cover changed stemming from land use change directly alter landscape properties, thereby affecting the CWRs population through cascading effects (da Silva and Hernández, 2014). For example, the change of land cover causes landscape composition changes. Thus, the change or disappearance of a biological community and the loss of the natural or seminatural habitat of wild populations eventually lead to population decline or even extinction of CWRs (Niedrist et al., 2009; Malavasi et al., 2018). The community and its shift, which is usually accompanied by land use change, have a great effect on wild populations (Newbold et al., 2015). Thus, we need a deep understanding of how land use change affects the communities surrounding CWRs populations.

The practices of biodiversity conservation, including those for CWRs, have been continuously moving forward. For example, the European Union (EU) has implemented sustainable enhancement, organic agriculture, and agricultural environment programs to reduce the negative impact of agricultural activities on biodiversity (Andersson et al., 2012; Batáry et al., 2015). Meanwhile, China has carried out the most extensive programs for ecosystem services worldwide, such as the Natural Forest Conservation Program (NFCP) and Grain-to-Green Program (GTGP; also known as the Farm-to-Forest Program), as well as important agricultural biological resource protection actions, to restore natural vegetation and protect biodiversity (Xu et al., 2006; Liu et al., 2008). Since the implementation of these programs, many national *in situ* and *ex situ* protection zones of CWRs have been established. Thus far, China has built 205 *in situ* conservation zones for 39 CWRs, including 65 *in situ* conservation zones for wild rice species. In particular, China established two national wild rice germplasm resource nurseries, where the plants of more than 17,000 wild rice germplasm resources are growing (Xu et al., 2020). Although these conservation practices greatly benefit the survival of wild populations of CWRs, the attention to the effects of these practices experiencing the impacts of land use change on the community surrounding the CWRs population and the comparison of *in situ* conserved populations with not conserved ones remain lacking.

Wild rice species are the wild relatives of cultivated rice (*Oryza sativa*; Zheng et al., 2019). They are also important germplasm resources for rice breeding because they contain abundant insect-resistant, stress-tolerant, high-yield, or/and male sterility genes. For instance, the utilization of male sterility genes from the common wild rice (*O. rufipogon*) generated the world-famous hybrid rice (*O. sativa*) (Liu et al., 2014). Three wild rice species are distributed in China: *O. rufipogon*, *O. officinalis*, and *O. granulata* (Dai et al., 2004). The three wild rice species mainly occur on the edges of farmlands and forest zones. Their habitats are constantly disturbed by land use changes mainly caused by agricultural activities, resulting in a serious decline in their population. The Chinese government has listed the three wild rice species as national class II protected species and launched two national surveys for wild rice resources (1978–1982 and 2001–2004; Xu et al., 2020). Consequently, dozens of natural populations of wild rice were conserved *in situ*. Moreover, the results of the two national surveys show that Chinese wild rice populations are still declining, even though great effort has been paid to wild rice population conservation and the surviving wild rice populations generally hold relatively rich genetic diversity (Yang et al., 2013). This population decline may be due to habitat destruction or loss caused by land use change. In addition, the three wild rice species display remarkable niche differentiation (Zhang and Yang, 2003). The three plants have specific habitat preferences and occur in different ecological systems. Moreover, their populations may face different land use changes due to agricultural activities. Therefore, the three wild rice species provide us an ideal system to compare the population survival statuses of CWRs with different typic or intensive disturbances and obtain general insights into the effects of land use change on CWRs.

The present study used the three wild rice species as a model, aims to examine the effects of land use change on the survival of wild populations. We performed another national wild rice population survey in 2019 based on the historical survey records (1978–2004). We investigated the influence of land use change on wild rice populations by using the data of population survival status combined with the Landsat Earth observation information of land use change in the recent 20 years (2001–2019), covering the range of the three wild rice species. This study aims to answer the following questions: (1) What is the habitat preference of wild rice species? (2) What kind of land use change have the three wild rice species experienced? (3) Do land use change and population decline in wild rice species have significant correlations? The obtained data provide not only direct guidelines for the conservation of wild rice but also new insights into the mechanisms underlying the influence of land use change on CWRs.

## Materials and methods

### Study area

The basic unit of the research object is the population in the ecological concept. We recorded the three wild rice species (*O. rufipogon*, *O. officinalis*, and *O. granulata*) with a geographical space interval of less than 300 m as the same population. The first national wild rice survey (1978–1982) comprehensively recorded the geographical distribution range of all wild rice populations in China (excluding Taiwan Province), showing that *O. rufipogon* is distributed in Jiangxi, Hunan, Yunnan, Guangxi, Guangdong, Fujian and Hainan Province; *O. officinalis* in Yunnan, Guangxi, Guangdong and Hainan Province; and *O. granulata* in Yunnan and Hainan Province (Supplementary Figure S1; The Cooperative Team of Wild Rice Resources Survey and Exploration of China, 1984; Xu et al., 2020). Subsequent studies are all based on these data of geographical distribution range and population status. The second national survey (2001–2004; Xu et al., 2020) covered the geographical range entirely as the first one. In the present study, the sampled populations were randomly selected from those survived in the second national survey and also cover the whole distribution range of the three wild rice species in China. GPS data were recorded at the center of the geographical range of each population.

### Population information

The data of the first national survey were from the survey reports (The Cooperative Team of Wild Rice Resources Survey and Exploration of China, 1984). The population data of the second national survey were obtained from the literature reports, survey reports, books, and other records of the Provincial Academy of Agricultural Sciences (Pang et al., 2000; Pang and Chen, 2002; Zhang and Yang, 2003; Gai et al., 2005; Xu et al., 2020). Some of the original recorded “populations” were combined and recorded as the same population when the distance between individual “populations” is less than 300 m, then we obtained the basic data of population survival status of the three wild rice species in 2001. To further monitor the population survival status of the three wild rice species in China recently, we conducted a filed survey across the entire range of the three wild rice species from 2016 to 2019. A total of 195 populations survived were investigated in our survey, including 118 *O. rufipogon*, 35 *O. officinalis*, and 42 *O. granulata* (Supplementary Tables S1–S3), in which wild rice plants still exist was recorded as present population; otherwise, it was noted as lost one. We used the data of population status in 2001 and 2019 to do land use change analysis.

### Land use type

Land use type is usually characterized as land cover type, such as farmland, water body, natural vegetation, and so on, that all are easily recorded by satellite (Zalles et al., 2021). Therefore, we can describe the land use change of wild rice habitats during a period based on the land cover data observed by satellite in different years. The satellite-observed land cover data were derived from GlobCover version 2.32009.^1^ These data are available as a raster with 23 land cover classes at a resolution of 300 m. The data include natural land cover and artificial land use types, which are uniformly recorded as land use in this study. We used land use types in grids of GPS points that were repositioned in the center of the population to obtain the land use types of the 195 populations. To obtain the data of the land use types of communities around the habitats of wild rice populations, we extracted all land use types of each population within a radius of 5 km centered on the GPS point and calculated the area of each land use type in the circle to represent the composition and area of the community surrounding the population. The information of four populations of *O. rufipogon* could not be extracted. Finally, we extracted the data of 191 populations and their surrounding communities. Among them, for *O. rufipogon,* there are 64 population in presence, and 50 population lost; for *O. officinalis*, there are 23 population in presence, and 12 population lost; for *O. granulata*, there are 31 population in presence, and 11 population lost (Supplementary Tables S1–S3). In this study, 12 land types were obtained. They comprise three types of TC (tree cover): tree cover, broadleaved, evergreen; tree cover, broadleaved, deciduous; tree cover, needle leaved, evergreen. The following are the other nine land types: cropland, rainfed (RC); cropland, irrigated, or postflooding (IC); mosaic cropland (MCN, > 50% natural vegetation, < 50%); mosaic natural vegetation (MNC, > 50% cropland, < 50%); mosaic tree and shrub (MTH, > 50% herbaceous cover, < 50%); shrubland (SL); tree cover, flooded, fresh, or brackish water (TCF); urban areas (UA); water bodies (WB). The extracted data also include NA, indicating no data. The data remained stable from 2000 to 2019, so we leave them out. The extraction of land use types of each population in satellite observation data was performed in R 4.0.1 using a self-written R script.

### Data analysis

The survey data in 1978–2019 that we used were divided into two periods: 1978–2000 and 2001–2019. We also calculated the loss proportion of wild rice populations in each period. The loss proportion of the population from 1978 to 2000 was obtained by comparing the second national survey data (2001–2004) with the first national survey data (1978–1982). The loss proportion of the population in 2001–2019 was obtained by comparing the populations survey data of 2016–2019 with the data of the second national survey (2001–2004).

We considered 4 years as the statistical unit to reduce the statistical error of the community area around the population. This statistical unit was divided into five periods: 2000–2003, 2004–2007, 2008–2011, 2012–2015, and 2016–2019. The average value of the 4-year data in each period represents this time. The period from 2000 to 2003 was used as the baseline, and the area proportion of each type in the other four periods was compared with the baseline to obtain the change proportion of the current period relative to the baseline period. In this study, in order to compare the difference of land use area between the present and lost populations, the significance test between the present population and the lost population (the area of each type within the 5 km range in 2001) was conducted in R 4.0.1 using Wilcoxon rank sum test.

## Results

### Survival dynamics of wild rice populations

**Figure 1:**
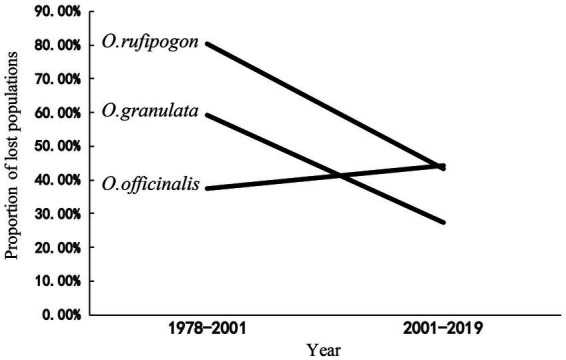
The proportion of lost populations in two period (1978–2001,2001–2019). The data of surveys in 1978–1982 as baseline, the proportion of loss population in 1978–2001 was compared with data of two national surveys 1978–1982 and 2001–2004; The proportion of lost populations in 2001–2019 was compared with data of national surveys (2001–2004) and our data in 2019.

After 20 years since the first national survey [1978–2000 (S1)], the survival rates of wild populations of *O. rufipogon*, *O. officinalis*, and *O. granulata* decreased to 20, 63, and 40%, respectively, when the land change was first clearly observed by satellites in 2001. Population decline for the three wild rice species has continued in the recent 20 years [from 2001 to 2019 (S2)], with population loss rates of 43.2, 44.3, and 26.2% (Figure 1). These findings indicate that the three wild rice populations have experienced a high-speed extinction trend in the past 40 years. The comparison of the loss proportions of the populations in the two periods indicated that the population loss proportion of *O. rufipogon* and *O. granulata* decreased considerably in the S2 period. This decrease was approximately 1/2 of that in the S1 period (80.3–43.2% and 59.3–26.2%). However, the population loss proportion of the *O. officinalis* population has accelerated (37.5–44.3%; Figure 1).

### Land use types of habitats of the three wild rice populations in 2001

**Figure 2:**
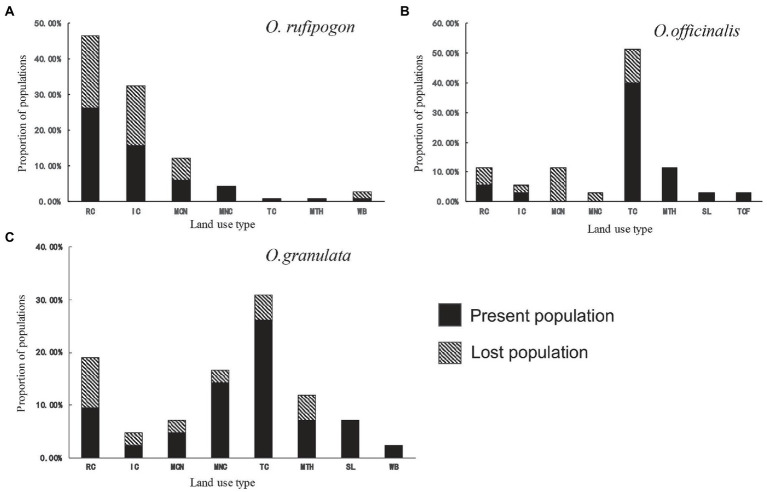
Population proportion per land use type of **(A)** *O. rufipogon* **(B)** *O. officinalis* **(C)** *O. granulata* during 2001~2019. Present population is the population survived during 2001~2019; Lost population presented in 2001 but disappeared in 2019. RC, rainfed cropland; IC, irrigated or postflooding cropland; MCN, mosaic cropland, >50% / natural vegetation, <50%; MNC, mosaic natural vegetation, >50% / cropland, <50%; TC, tree cover; MTH, mosaic tree and shrub, >50% / herbaceous cover, <50%; SL, shrubland; TCF, tree cover, flooded, fresh, or brackish water; WB, water bodies.

The land use types of the three wild rice population habitats are basically the same in 2001 (Figure 2). They include the land use types of RC, IC, MCN, MNC, TC, and MTH, but their proportions vary greatly. The proportion of farmland (RC and IC) and mosaic farmland (MCN and MNC) in the habitat of *O. rufipogon* populations is 94.7% (Figure 2A; Supplementary Table S4). The proportions of either type in the habitats of *O. officinalis* (31.4%) and *O. granulata* (47.6%) are low (Figures 2B,C; Supplementary Table S4). The proportion of TC in the habitats of *O. officinalis* and *O. granulata* is the highest, accounting for 51.4 and 31.0%, respectively; however, it is less than 1% in the habitat of *O. rufipogon*. This finding shows that obvious habitat preference differences exist among the three wild rice species.

Further analyses of the habitat types of the lost populations from 2001 to 2019 showed that the populations whose habitats were in the farmland system (RC, IC, MCN, and MNC) reached a high loss proportion: 96% of the lost population habitats of *O. rufipogon* (Figure 2A) and 66.7% of the lost population habitats of *O. officinalis* (Figure 2B) and *O. granulata* (Figure 2C) are in the farmland system. This finding suggests that the populations in agricultural systems are under a great threat from agricultural activities.

### Composition and dynamics of communities around the habitat of the three wild rice populations

**Figure 3:**
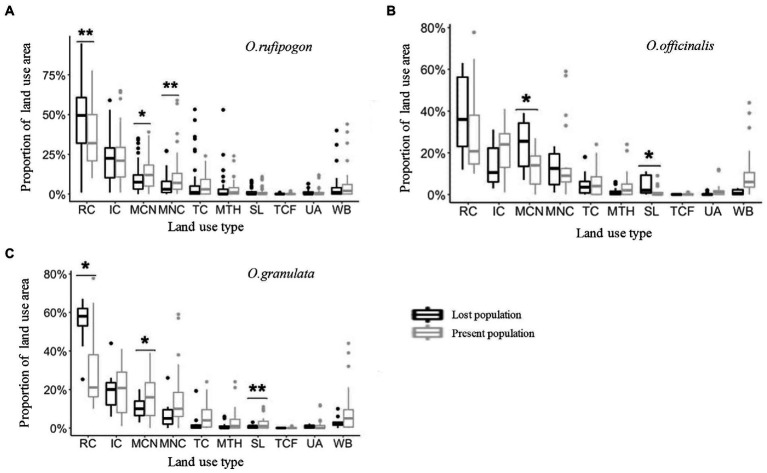
The land use area proportion in **(A)** *O. rufipogon* **(B)** *O. officinalis* **(C)** *O. granulata* within a radius of 5 km during 2001~2019. Present population is the population survived during 2001~2019; Lost population presented in 2001 but disappeared in 2019. ^*^p < 0.05, and ^**^p < 0.01 (Wilcoxon rank sum test). RC, rainfed cropland; IC, irrigated or postflooding cropland; MCN, mosaic cropland, >50% / natural vegetation, <50%; MNC, mosaic natural vegetation, >50% / cropland, <50%; TC, tree cover; MTH, mosaic tree and shrub, >50% / herbaceous cover, <50%; SL, shrubland; TCF, tree cover, flooded, fresh, or brackish water; UA, urban areas; WB, water bodies.

The land use types of communities around the habitats of the three wild rice populations include 10 types: RC, IC, MCN, MNC, TC, MTH, SL, TCF, UA, and WB. However, the area proportion varies greatly. The communities around the habitats of the three wild rice populations mainly comprise farmland systems (RC, IC, MCN, and MNC; Figure 3). The results show that the present population of *O. rufipogon* has significantly low RC (Wilcoxon rank sum test, *p* = 0.0047) area proportion and significantly high MCN (*p* = 0.0338) and MNC (*p* = 0.0036) area proportion (Figure 3A; Table 1). The present population of *O. officinalis* has a significantly low area proportion of MCN (*p* = 0.0106) and SL (*p* = 0.0207; Figure 3B; Table 1). The present population of *O. granulata* has a significantly low area proportion of RC (*p* = 0.0402), and significantly high area proportion of MCN (*p* = 0.0149) and SL (*p* = 0.0014; Figure 3C; Table 1). Compared with the lost populations, the communities around the habitat have a significantly high proportion of vegetation area, indicating the potential protective role of vegetation in the survival of the three wild rice populations.

**Table 1:** *p*-Values generated from Wilcoxon Rank Sum test testing the difference of land use types area around populations.

Species		RC	IC	MCN	MNC	TC	MTH	SL	TCF
*O. rufipogon*	W	1121.5	1,627	1994	2,132	1725.5	1783.5	1,654	1725.5
	P	0.0047	0.9706	0.0338	0.0036	0.5432	0.3301	0.8339	0.1235
*O. officinalis*	W	90.5	183.5	64	134.5	143	166.5	75	162
	P	0.1022	0.1171	0.0106	0.9167	0.8725	0.3054	0.0207	0.1385
*O. granulata*	W	78.5	164.5	67.5	132	143	166.5	50	168
	P	0.0402	0.3654	0.0149	0.8479	0.8736	0.3055	0.0014	0.0909

RC, rainfed cropland; IC, irrigated or postflooding cropland; MCN, mosaic cropland, > 50% /natural vegetation, < 50%; MNC, mosaic natural vegetation, > 50% /cropland, < 50%; TC, tree cover; MTH, mosaic tree and shrub, > 50% /herbaceous cover, < 50%; SL, shrubland; TCF, tree cover, flooded, fresh, or brackish water. W, Wilcoxon Rank sum test statistics; *p*, *p*-values.

The dynamics of the communities around the populations from 2001 to 2019 were analyzed further (Supplementary Figures S2–S4). The area proportion of farmland systems (RC and IC) around almost all populations of the three wild rice species showed a gradually decreasing trend in four periods (2004–2007, 2008–2011, 2012–2015, and 2016–2019), whereas the area proportion of the natural communities (TC, MTH, SL and TCF) showed an increasing trend. This observation shows that the area of farmland around the population has been decreasing and the area of vegetation has been increasing in the recent 20 years. The proportion of mosaic farmland (MCN and MNC; Supplementary Figures S3C,D) in some populations for *O. officinalis* has shown a considerable decline in lost populations relative to the present populations, whereas the proportions of TC and SL for *O. officinalis* have shown a considerable increase (Supplementary Figures S3E,G) in the recent period (2016–2019). This finding suggests that the transformation of mosaic farmland (MNC and MCN) to natural vegetation (TC and SL) has occurred in the recent period.

## Discussion

Monitoring habitat change and evaluating its effect on population survival help in understanding the impact of land use change on biodiversity (Fahrig et al., 2011; Kennedy et al., 2013; Zabel et al., 2019; Zalles et al., 2021). The present study evaluated the influence of land use change on the population survival of the three wild rice species in China for the first time through integrative analysis of the data on population survival in the recent 40 years and the remote sensing data of land use change across the range of wild rice distribution in the recent 20 years. The populations of the three wild rice species are affected by different land use change depending on their specific habitat preference. *O. rufipogon*, which constantly exists in farmland systems, suffers the most consequential impacts of land use change. The natural vegetation surrounding wild populations can considerably mitigate the destructive effect of land use change on the habitat of wild rice species, allowing the refuge of wild populations.

The combined analyses of historical records of population distribution and our field investigation data show that the three wild rice species are distributed inconsistently, resulting in populations isolated from one another. Although their habitat land cover types are basically the same, the proportion of each cover type considerably varies (Dai et al., 2004; Yang et al., 2013). *O. rufipogon* is mainly distributed in the shallow water or wet regions of inland swamps, such as ponds, swamps, streams, and riversides in hills and plains. The land cover types of its habitat are mainly farmland (RC and IC) and mosaic farmland (MCN and MNC; Figures 2, 3), resulting in its populations being often nearby rice fields (Song et al., 2003), and suggesting the similar habitat requirements between *O. rufipogon* and cultivated rice. *O. officinalis* is a hydrophilic plant and commonly distributed in mountainous streams, puddles, and other humid areas. The land cover type of its population is mainly forest (Figure 2). *O. granulata* is a xerophyte and understory plant. It is often found in the gaps of shrub and arbor forests on the hillside. The land cover type of its habitat is also mainly forest (Figure 2). These results demonstrate that the land cover types of population habitats are different between the three rice species and the habitat preferences of the three rice species are specific. This pattern is related to niche differentiation during speciation.

The analysis of land use change reveals that the three wild rice species have experienced changes in habitats (land cover types) with different extents. The populations of *O. rufipogon* are usually scattered in farmland systems with little space for reclamation (wasteland or barren land). Thus, *O. rufipogon* shows a relatively stable state (Supplementary Figure S5A). Conversely, the populations of *O. officinalis* and *O. granulata*, commonly occurring in seminatural or natural systems nearby farmland systems, face a serious threat of habitat change (Supplementary Figures S5B,C). This pattern hints that land use change is much intensive at the edge of the farmland system or forest edge (Bryant et al., 2020). Further analysis of the community (land cover area) around the population shows that the populations of the three wild rice species have a similar trend of community shift, with a slightly decreased area of farmland and increased area of natural vegetation (Supplementary Figures S2–S4). This pattern may be attributed to the implementation of NFCP and GTGP (Liu et al., 2008; Deng et al., 2014).

We further analyzed the relationship between land use change and the population decline of wild rice species. The population numbers of the three wild rice species have considerably varied since the first survey in the 1980s (Figure 1). This population decline is obviously caused by habitat development or/and destruction (Dai et al., 2004). We found that the survival rates vary between the wild rice populations occurring in different habitats (different land cover types and type proportions) and almost all the lost populations of the three wild rice species come from the farmland system, and the lost rate of populations in other habitats is very low (Figure 2). These results can be explained by the fact that the habitat of wild rice is directly occupied because of land use change or/and habitat destruction due to agricultural activities, such as the use of chemical fertilizers, pesticides, and herbicides (Tilman et al., 2001). It supports the view that the change in agricultural practices strongly affects the biodiversity in the farmland ecosystem (Sala et al., 2000; Titeux et al., 2016; Ellis, 2019). The populations of *O. officinalis* and *O. granulata* are influenced by other types of land use (Figure 2), such as abandonment of farmland or orchard that leads to intensive interspecific competition due to weed spring up (Matus et al., 2003; Deák et al., 2011).

The status of wild rice populations (including population stability and survival) in different habitats is correlated with the area of their surrounding vegetation. Our field investigation showed that dozens of *in situ* conserved populations, including the three wild rice species, grow well; however, the community type and the proportion of different land cover types of these conserved populations seem to be similar to those of populations that disappeared (Supplementary Figure S6). The reason is that human disturbance is effectively avoided by physical barriers, such as fences or wire entanglements, or mitigated by the buffer zone around the conserved populations (Gonçalves-Souza et al., 2021). For the un-conserved populations, those with a small area of natural vegetation exhibit high extinction rates (Figure 3). The lower the area of vegetation/farmland surrounding wild rice populations is, the lower the landscape heterogeneity is, and the weaker the ecological flexibility of wild populations is (Niedrist et al., 2009; Malavasi et al., 2018; Newbold, 2018). This finding indicates that natural vegetation can effectively mitigate the impacts of human activities on wild populations. Playing the role of a “biological barrier” (Santoro et al., 2012), natural vegetation can buffer the pressure of herbivore grazing and trampling and the spillover effect of pesticide spraying and chemical fertilizer on wild rice. This “biological barrier” effect coincides with the good population survival of *O. rufipogon* and *O. granulata*, following the increase of natural vegetation since the implementation of natural ecological programs (Liu et al., 2008). Thus, the present study agrees with the view that, like physical barriers, natural vegetation plays the role of a “biological barrier” to alleviate the impacts of land use change on wild populations (Reyers et al., 2010).

With the Landsat Earth observation information of land use change and the field population investigation, the present study reveals that the habitats of three wild rice populations are constantly affected by land use change due to human activities, particularly agricultural activities. Our findings suggest that remote sensing technology can be used effectively to monitor population status, land use changes, and community around wild population in real time and quantify the role of vegetation type and area in buffering the impact of human activities. This study also aims to consider the composition of communities around wild populations as a critical component of the assessment system in monitoring the population dynamics of wild rice species. In addition, our results call for the management and limitation of human agricultural activities, such as the use of herbicides, the expansion of cultivated lands, and grazing, to reduce the impact of agriculture on community integrity and improve the resilience and sustainability of the ecosystem of sympatric wild relative species. Generally, the present study highlights the biological barrier role of natural vegetation surrounding wild populations in suffering from the destructive effect of land use change on wild rice species. To further understanding the effects of land use change on CWRs, we need more studies monitoring the dynamics of population and its surrounding vegetations by using remote sensing technology.

## Data availability statement

The original contributions presented in the study are included in the article/Supplementary material, further inquiries can be directed to the corresponding author.

## Author contributions

ZS, YW, WZ, LL, and JY conceived the study. HC, SD, ZH, YC, DT, and YL collected the data. HC performed the statistical analyses and wrote the first draft of manuscript. All authors contributed to the article and approved the submitted version.

## Funding

The study was supported by the National Natural Science Foundation of China (31570383) and the Biodiversity Survey, Observation and Assessment Program of Ministry of Ecology and Environment of China (2019-5-12).

## Conflict of interest

The authors declare that the research was conducted in the absence of any commercial or financial relationships that could be construed as a potential conflict of interest.

## Publisher’s note

All claims expressed in this article are solely those of the authors and do not necessarily represent those of their affiliated organizations, or those of the publisher, the editors and the reviewers. Any product that may be evaluated in this article, or claim that may be made by its manufacturer, is not guaranteed or endorsed by the publisher.

## Footnotes

^1^ http://due.esrin.esa.int/page_globcover.php
