# Supplementary material for: Effects of land use change on population survival of three wild rice species in China since 2001
Source: Front Plant Sci. 2022 Sep 6;13:951903. doi: 10.3389/fpls.2022.951903 (PMC9488966; doi:10.3389/fpls.2022.951903)
Supplement: Supplementary file 1 [file Data_Sheet_1.PDF]

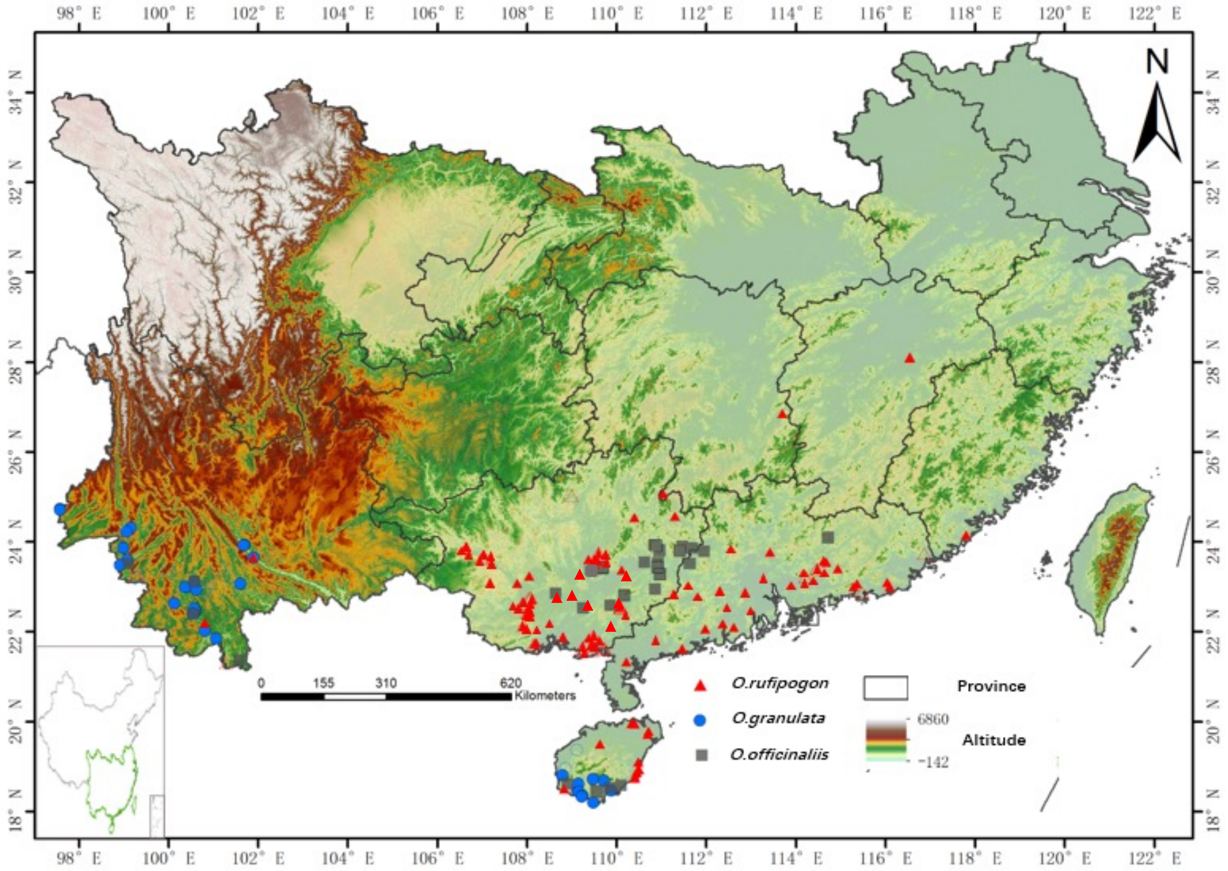

Fig. S1. The geographical distribution range of all wild rice populations in China in 1978. Data from The Cooperative Team of Wild Rice Resources Survy and Exploration of China., 1984.

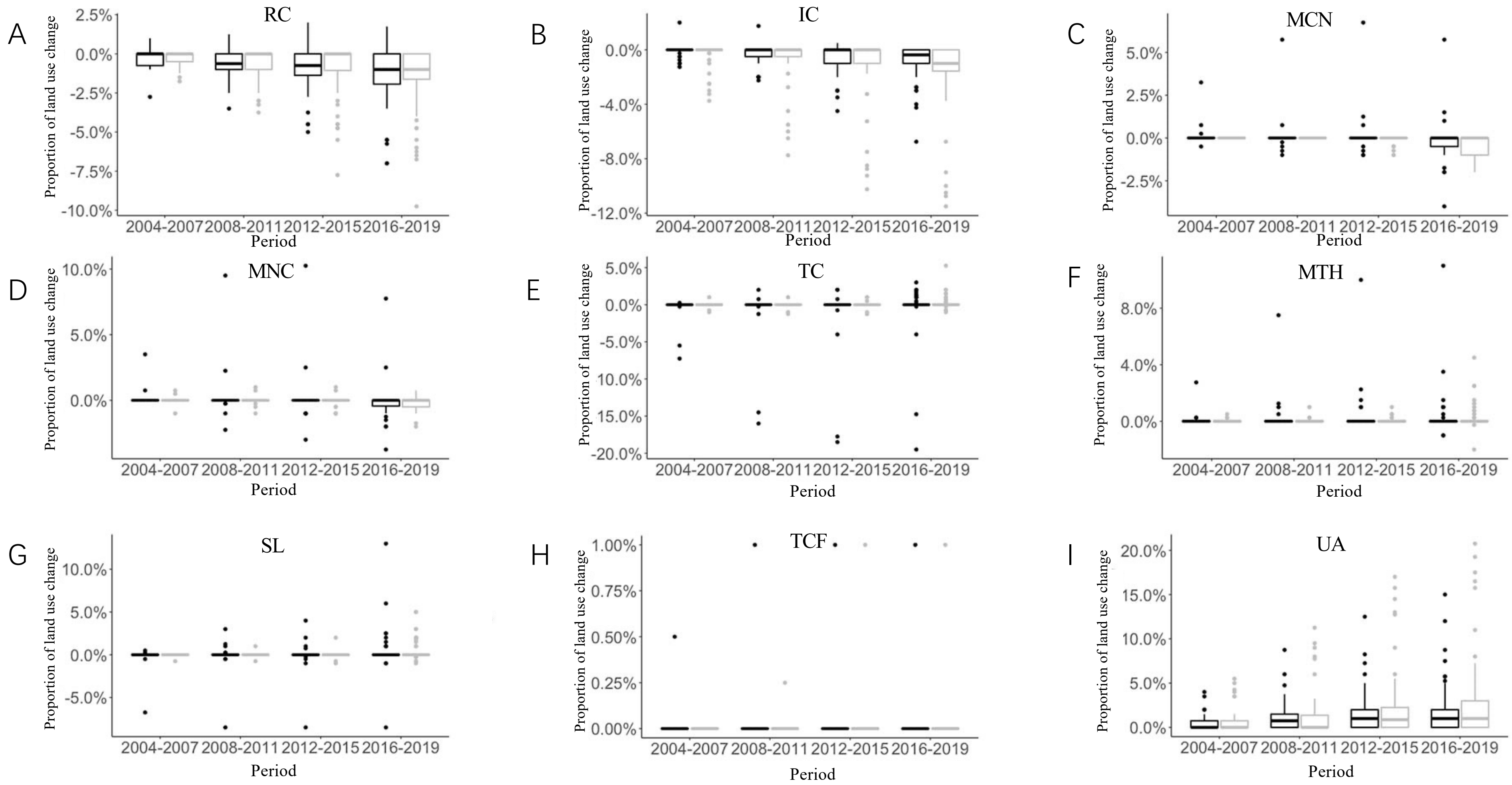

A
RC
B
IC
C
MCN
D
MNC
E
TC
F
MTH
G
SL
H
TCF
I
UA
Proportion of land use change
Period
2004-2007
2008-2011
2012-2015
2016-2019

Fig. S2.

J

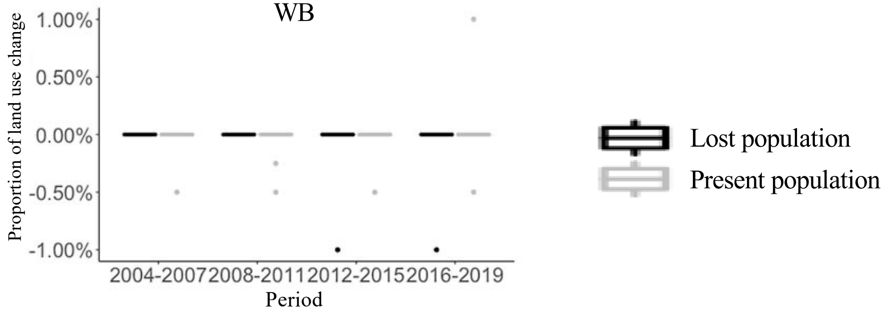

Fig. S2. The change rate of each land use types (A) RC (B) IC (C) MCN (D) MNC (E) TC (F) MTH (G) SL (H) TCF (I) UA (J) WB of *Oryza rufipogon* population in four period (2004-2007,2008-2011,2012-2015,2016-2019) , 2001-2003 as the base line. Present population is the population survived during 2001~2019; Lost population presented in 2001but disappeared in 2019. RC, rainfed cropland; IC, irrigated or postflooding cropland; MCN, mosaic cropland, >50% / natural vegetation, <50%; MNC, mosaic natural vegetation, >50% / cropland, <50%; TC, tree cover; MTH, mosaic tree and shrub, >50% / herbaceous cover, <50%; SL, shrubland; TCF, tree cover, flooded, fresh, or brackish water; UA, urban areas; WB, water bodies.

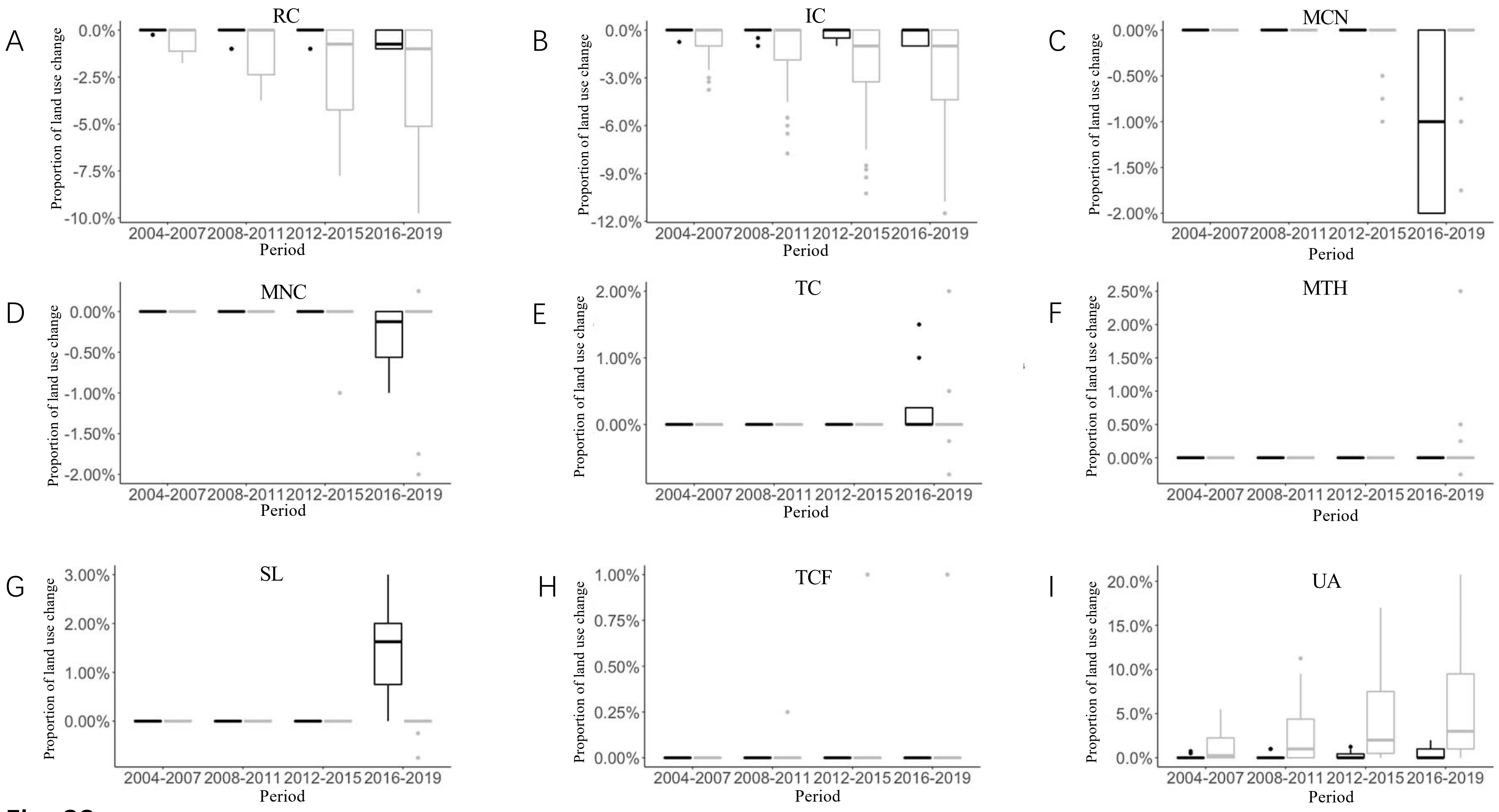
A
RC
B
IC
C
MCN
D
MNC
E
TC
F
MTH
G
SL
H
TCF
I
UA
Proportion of land use change
Period
2004-2007
2008-2011
2012-2015
2016-2019
0.0%
-2.5%
-5.0%
-7.5%
-10.0%
-3.0%
-6.0%
-9.0%
-12.0%
0.00%
-0.50%
-1.00%
-1.50%
-2.00%
1.00%
2.00%
0.50%
1.50%
2.50%
3.00%
0.25%
0.75%
5.0%
10.0%
15.0%
20.0%

Fig. S3.

J

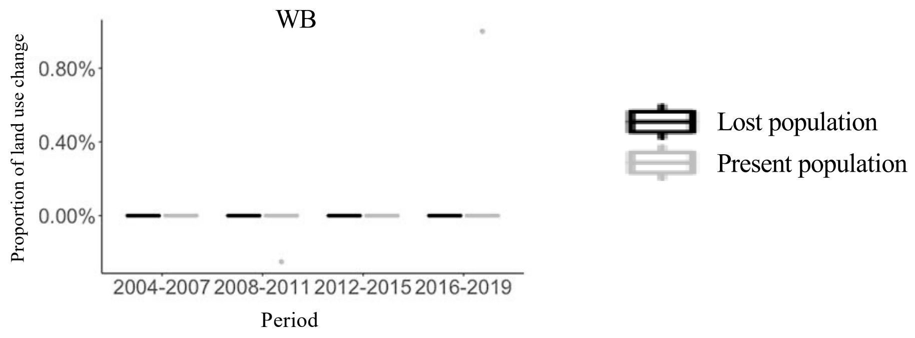

Fig. S3. The change rate of each land use types (A) RC (B) IC (C) MCN (D) MNC (E) TC (F) MTH (G) SL (H) TCF (I) UA (J) WB of *Oryza officinalis* population in four period (2004-2007,2008-2011,2012-2015,2016-2019) , 2001-2003 as the base line. Present population is the population survived during 2001~2019; Lost population presented in 2001but disappeared in 2019. RC, rainfed cropland; IC, irrigated or postflooding cropland; MCN, mosaic cropland, >50% / natural vegetation, <50%; MNC, mosaic natural vegetation, >50% / cropland, <50%; TC, tree cover; MTH, mosaic tree and shrub, >50% / herbaceous cover, <50%; SL, shrubland; TCF, tree cover, flooded, fresh, or brackish water; UA, urban areas; WB, water bodies.

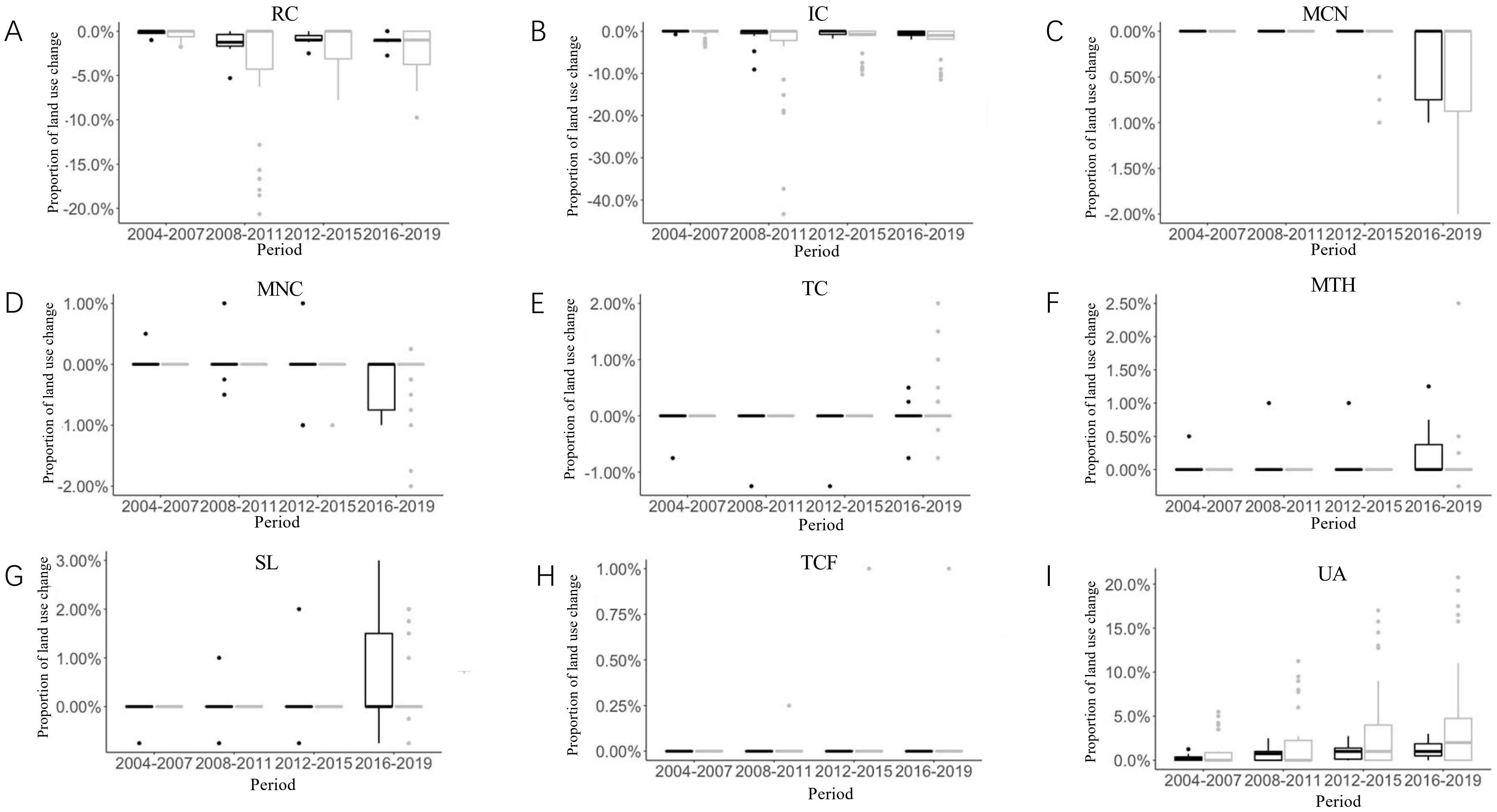

A
RC
0.0%
-5.0%
-10.0%
-15.0%
-20.0%
B
IC
0.0%
-10.0%
-20.0%
-30.0%
-40.0%
C
MCN
0.00%
0.50%
1.00%
1.50%
-2.00%
D
MNC
1.00%
0.00%
-1.00%
-2.00%
E
TC
2.00%
1.00%
0.00%
-1.00%
F
MTH
2.50%
2.00%
1.50%
1.00%
0.50%
0.00%
G
SL
3.00%
2.00%
1.00%
0.00%
H
TCF
1.00%
0.75%
0.50%
0.25%
0.00%
I
UA
20.0%
15.0%
10.0%
5.0%
0.0%
Proportion of land use change
2004-2007
2008-2011
2012-2015
2016-2019
Period

Fig. S4.

J

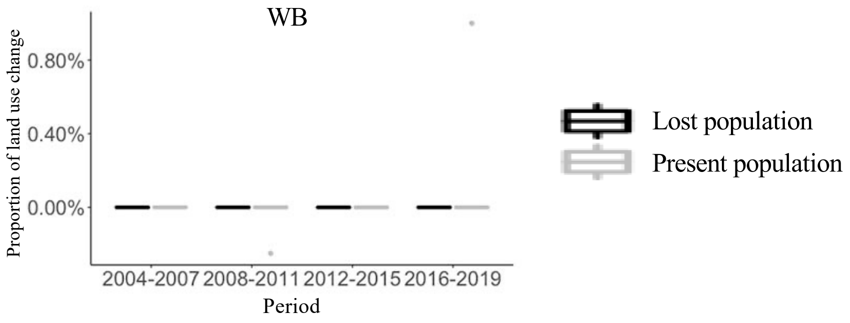

Fig. S4. The change rate of each land use types (A) RC (B) IC (C) MCN (D) MNC (E) TC (F) MTH (G) SL (H) TCF (I) UA (J) WB of *Oryza granulata* population in four period (2004-2007,2008-2011,2012-2015,2016-2019) , 2001-2003 as the base line. Present population is the population survived during 2001~2019; Lost population presented in 2001but disappeared in 2019. RC, rainfed cropland; IC, irrigated or postflooding cropland; MCN, mosaic cropland, >50% / natural vegetation, <50%; MNC, mosaic natural vegetation, >50% / cropland, <50%; TC, tree cover; MTH, mosaic tree and shrub, >50% / herbaceous cover, <50%; SL, shrubland; TCF, tree cover, flooded, fresh, or brackish water; UA, urban areas; WB, water bodies.

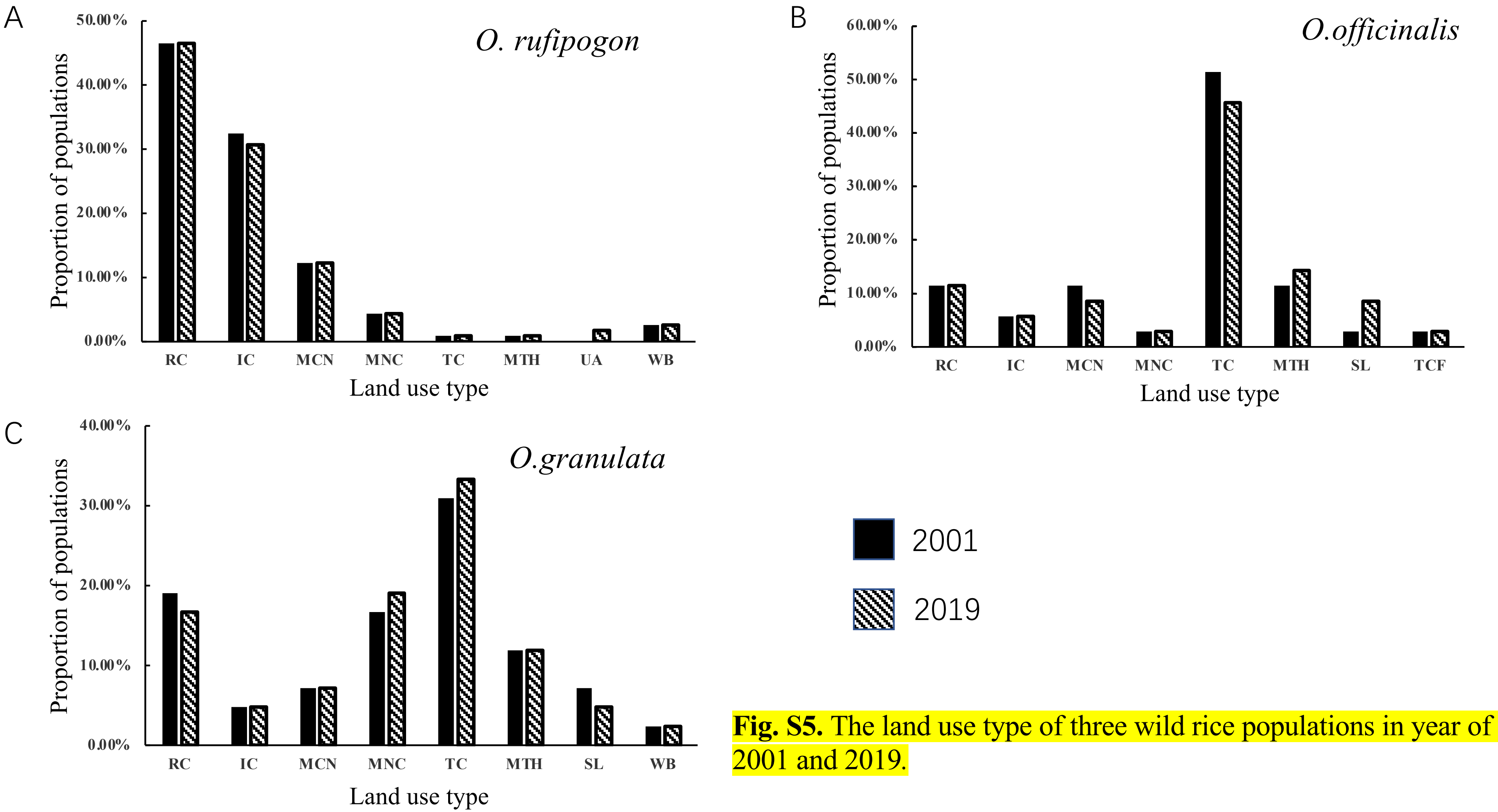

Fig. S5. The land use type of three wild rice populations in year of 2001 and 2019.

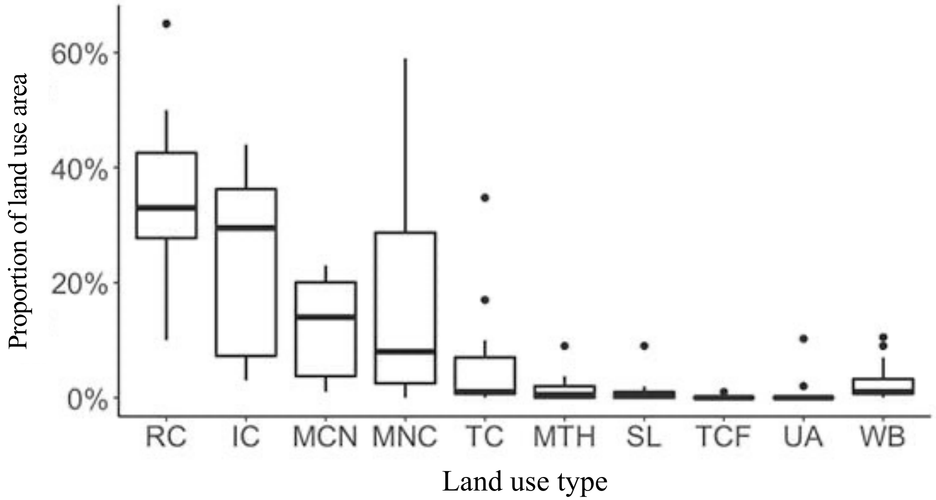

Fig. S6. The area proportion of *in situ* conserved *O. rufipogon* populations land use types within a radius of 5 km in year of 2001**.**
